# Diagnostic Performance of LI-RADS Version 2018 for Primary Liver Cancer in Patients With Liver Cirrhosis on Enhanced MRI

**DOI:** 10.3389/fonc.2022.934045

**Published:** 2022-07-01

**Authors:** Xinai Liu, Xiaoyan Ni, Yubo Li, Chun Yang, Yi Wang, Chunzheng Ma, Changwu Zhou, Xin Lu

**Affiliations:** ^1^ Department of Magnetic Resonance Imaging (MRI), Henan Provincial Hospital of Traditional Chinese Medicine (The Second Affiliated Hospital of Henan University of Traditional Chinese Medicine), Zhengzhou, China; ^2^ Department of Radiology, Zhongshan Hospital, Fudan University, Shanghai, China; ^3^ Shanghai Institute of Medical Imaging, Shanghai, China; ^4^ Department of Cancer Center, Zhongshan Hospital, Fudan University, Shanghai, China; ^5^ Department of Oncology, Henan Provincial Hospital of Traditional Chinese Medicine, Zhengzhou, China

**Keywords:** hepatocellular carcinoma (HCC), primary liver cancer (PLC), cirrhosis, LI-RADS, combined hepatocellular-cholangiocarcinoma (cHCC-CC), intrahepatic cholangiocarcinoma (ICC)

## Abstract

**Purpose:**

The study evaluated the diagnostic performance of the Liver Imaging Reporting and Data System (LI-RADS) version 2018 for differentiating hepatocellular carcinoma (HCC) from primary liver cancer in patients with liver cirrhosis based on the updated 2019 WHO classification.

**Materials and Methods:**

From 2016 to 2021, 300 patients with surgically confirmed primary liver cancer (PLC) and liver cirrhosis based on the updated 2019 WHO classification were eligible for this retrospective study (100 cases in each of three groups including HCC, ICC, and cHCC-CCA). Two radiologists were blinded to the final diagnosis and independently assigned an LI-RADS category to each liver nodule. The diagnostic performances of the LR-5 category (definitely HCC), and the LR-M category (probably or definitely malignant, but not specific for HCC) were calculated in overall and small observations (<20 mm). Comparisons between groups of categorical variables were performed by one-way analysis of variance and the Chi-squared or Fisher’s exact test.

**Results:**

The mean age of 300 patients (226 men and 74 women) was 57.40 ± 11.05 years. The sensitivity and specificity of the LR-5 category for differentiating HCCs from other primary liver cancers were 81% (81 of 100) and 82% (164 of 200), respectively. The LR-M category had a sensitivity of 63% (126 of 200) for diagnosing non-HCCs (ICCs and cHCC-CCAs), with a specificity of 90% (90 of 100). The LR-5 category had a sensitivity of 82.5% (33 of 40) for diagnosing HCCs in small observations (<20 mm) with a specificity of 76.6% (59 of 77). On the contrary, LR-M demonstrated slightly higher specificity (93.8%) and sensitivity (73.8%) for diagnosing non-HCCs with tumor size <20 mm.

**Conclusion:**

The LR-5 category as well as the LR-M category of Liver Imaging Reporting and Data System (LI-RADS) version 2018 can effectively distinguish hepatocellular carcinoma from other primary hepatic malignancies in patients with liver cirrhosis, especially for small observations (<20 mm).

## Introduction

Primary liver cancer (PLC) is the fourth most common malignant tumor and the second leading cause of cancer-related death worldwide (1, 2), which seriously threatens the life and health of cirrhotic patients. Primary liver cancer mainly includes three pathological types: hepatocellular carcinoma (HCC), intrahepatic cholangiocarcinoma (ICC), and combined hepatocellular-cholangiocarcinoma (cHCC-CCA). Compared with other liver malignancies that require pathological confirmation, HCC can be diagnosed by non-invasive preoperative imaging features in at-risk patients (3).

The Liver Imaging Reporting and Data System (LI-RADS), as a comprehensive system for standardizing the terminology, technique, interpretation, reporting, and data collection of liver imaging in patients at risk of HCC, is being increasingly adapted by many academic and nonacademic clinical practices (4, 5). The latest v2018 LI-RADS has been released by adjusting the LR-5 criteria and threshold growth definition (size increase of a mass by 50% in 6 months) to be concordant with the American Association for the Study of Liver Diseases (AASLD) guidance for the definite diagnosis and management of HCC (6, 7). Despite the low incidence of ICC and cHCC-CCA, when ICC and cHCC-CCA exhibit similar risk factors to HCC, including cirrhosis and chronic viral hepatitis (8, 9), the distinction between non-HCC and HCC is important in terms of treatment and prognosis (6).

A recent investigation demonstrated the performance of LI-RADS 2017 for diagnosing HCC in patients with cirrhosis on MRI with gadoxetate disodium contrast agent (10). Although several studies (6, 7, 11, 12) have examined the performance of LI-RADS 2018 in patients at risk of HCC, these studies have yielded inconsistent results and were hampered by small patient populations and gadoxetate disodium contrast agent. Moreover, with the continuous feedback of user experience, the 2019 WHO classification (13) revised the definition and category of combined hepatocellular cholangiocarcinoma (cHCC-CCA) (14). The 2019 WHO classification (13) revised the diagnostic term for biphenotypic PLCs by including a list of all tumor components (15) and abandoned subtype classifications of typical HCCs or CCAs expressed by immunohistochemical stem cell markers and cholangiolocarcinomas (CLC) without an HCC component in subtypes with stem cell features (14).

Therefore, the main purpose is to evaluate the performance of LI-RADS 2018 for primary liver cancer in the background of liver cirrhosis on MRI combined with extracellular contrast agents based on the updated 2019 WHO classification and whether its efficacy also applies to small liver tumors (<20 mm).

## Materials and Methods

### Study Population

**Figure 1:**
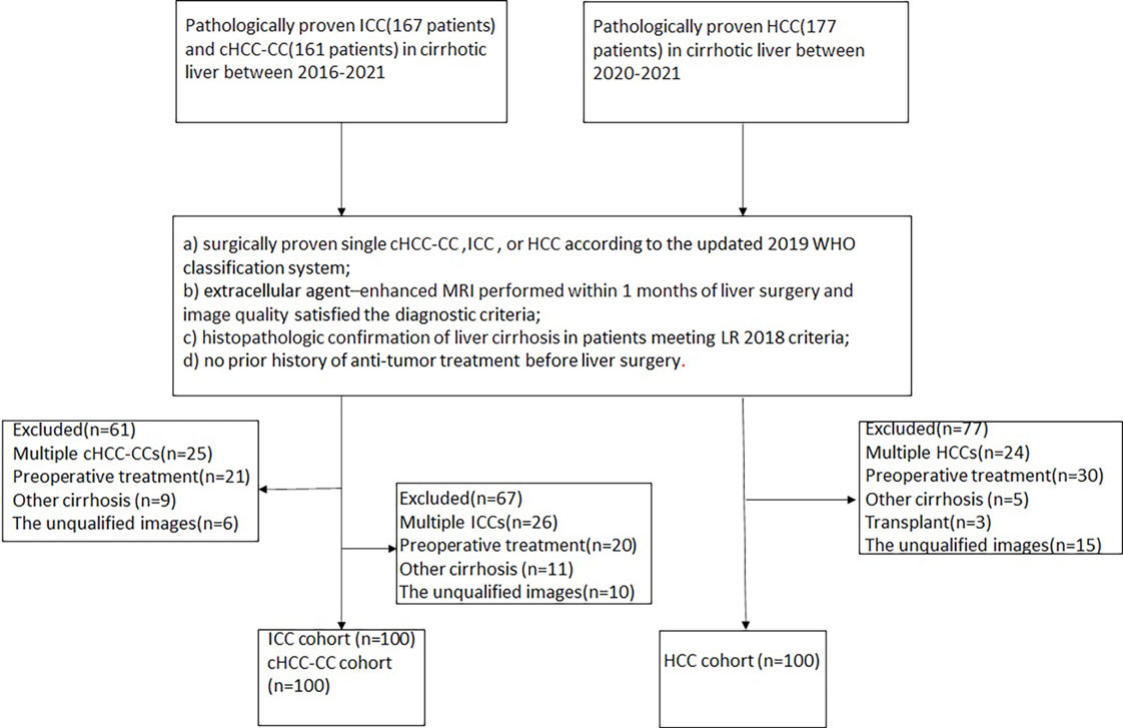
Flowchart of the study cohort. HCC, hepatocellular carcinoma; ICC, intrahepatic cholangiocarcinoma; cHCC-CCA, combined hepatocellular carcinomacholangiocarcinoma.

This retrospective study was approved by the institutional review board of our institution with a waiver of the written informed consent requirement. From January 2016 to November 2021, patients with either cHCC-CCA or ICC who met the following criteria were enrolled: a) surgically proven single cHCC-CCA or ICC according to the updated 2019 WHO classification system; b) extracellular agent–enhanced MRI performed within 1 month of liver surgery and image quality satisfied the diagnostic criteria; c) histopathologic confirmation of liver cirrhosis in patients meeting the LR 2018 criteria; and d) no prior history of anti-tumor treatment before liver surgery. HCC patients meeting the above categories were selected at random during the latter 1 year of the study period (between 2020 and 2021). Ultimately, 300 patients were enrolled in this study, namely, 100 HCC, 100 ICC, and 100 cHCC-CCA patients. All patients had Child–Pugh class A cirrhosis. The flowchart of patient enrollment is provided in **Figure 1**.

### Clinical Data Evaluation

The clinical data of the patients were retrospectively analyzed, namely, age, gender, HBV infection status, serum alpha-fetoprotein within 7 days before surgery (alpha fetoprotein, AFP), carcinoembryonic antigen (carcinoembryonic antigen, CEA) and carbohydrate antigen 19-9 (carbohydrate antigen 19-9, CA19-9), the critical value of AFP, CEA, and CA19-9. The cutoff values were 20 ng/ml, 5 ng/ml, and 37 U/ml, respectively.

### MRI Acquisition

All patients were scanned with a 1.5-T MR scanner (uMR 560, United Imaging Healthcare) equipped with a 24-channel body array coil. Routine liver protocols consist of transverse T2-weighted imaging with fat-suppressed fast spin-echo sequence (T2WI), T1WI breath-hold in-phase and opposed-phase sequences, and diffusion-weighted imaging (DWI, b value = 0, 50, and 500 s/mm^2^). Dynamic imaging was performed using a T1-weighted fat-suppressed breath-hold sequence. The arterial phase was acquired at 10–14 s when the contrast agent (gadolinium diethylenetriamine pentaacetic acid, Gd-DTPA; Magnevist, Bayer HealthCare) was administered in a vein at a dose of 0.1 mmol/kg at a rate of 2 ml/s. The portal venous phase and delayed phase sequences were acquired at 70–90 s and 160–180 s, respectively.

### Image Feature Analysis

All MR images were retrospectively evaluated by two radiologists (CWZ and CY, who have 12 and 14 years of experience in abdominal imaging, respectively), who were blinded to the specific results of the pathological examination but were informed of the presence of tumor. They independently assigned an LI-RADS category to each nodule. All observers had access to all imaging studies through picture archiving and communication systems (PACS) from our institutions. A consensus was reached by discussion when there was disagreement between the two observers.

### LI-RADS Major Features and Targetoid Mass Features

The following imaging characteristics of lesions were investigated on MR images according to LI-RADS version 2018 (8): nodule size, location, and presence or absence of major features (nonrim arterial phase hyperenhancement (APHE), non-peripheral washout, and enhancing capsule), and targetoid mass features (APHE), peripheral washout, delayed central enhancement, and targetoid restriction).

### Ancillary Imaging Features

The ancillary imaging features in our study combined a part of LI-RADS 2018 ancillary and additional features (particularly prone to malignancy, not HCC) not included in the LI-RADS 2018 algorithm, which are beneficial for differential diagnosis according to the available literature (16, 17) and our clinical experience. The following ancillary imaging features favoring HCC were evaluated: a) nodule-in-nodule: smaller nodule with different imaging signs appeared in the mass; b) mosaic architecture: the interior of the mass was divided into multiple heterogeneous nodules or compartments, usually showed heterogeneous signal on T2WI; c) fat in mass: signal intensity reduced on T1WI opposed-phase images versus T1WI in-phase images; and d) blood products in mass: hyperintense on T1WI in- and opposed-phase images and hyperintense or heterogeneous on T2WI. Additionally, these other ancillary features favoring malignancy, not HCC in particular, based on the literature and experience were also evaluated: a) restricted diffusion: intensity on DWI, not attributable solely to T2 shine-through; b) mild-moderate T2 hyperintensity; c) peritumoral biliary dilatation; d) liver surface retraction: the liver surface was retracted by adjacent tumors; and e) corona enhancement: peri-observational enhancement in late arterial phase or early PVP attributable to venous drainage from tumor.

The radiologists assigned a LI-RADS category to each nodule. Nodules were classified as category LR-3 (intermediate probability of HCC), LR-4 (probably HCC), LR-5 (definitely HCC), LR-M (probably malignant, not specific for HCC), or LR-TIV (nodule with a definite tumor in the vein). Threshold growth was excluded because our study had only one preoperative MRI examination.

### Statistical Analysis

Statistical analyses were performed using SPSS 26.0 (IBM). Continuous variables were expressed as mean ± standard deviation or median (interquartile range), while categorical variables were expressed as numbers and percentages. Inter-observer agreement of categorical variables was estimated by calculating Cohen’s kappa statistics (0.0–0.2, poor; 0.2–0.4, fair; 0.4–0.6, moderate; 0.6–0.8, substantial; 0.8–1.0, perfect). Comparisons between groups of categorical variables were performed by one-way analysis of variance and the Chi-squared or Fisher’s exact test.

## Results

### Patient Clinical Data

The clinical characteristics of the 300 patients (226 men [mean age, 56.69 ± 11.21 years] and 74 women [mean age, 59.57 ± 10.34 years]; overall mean age, 57.40 ± 11.05 years) are summarized in **Table 1**. The cHCC-CCA cohort had a younger age and a smaller size than the ICC and HCC cohorts, and the differences were statistically significant (P = 0.001, 0.017, respectively). In all three groups, hepatitis B virus was the most common cause of liver cirrhosis. The CA19-9 >37 U/ml was more often in the ICC patients than in the cHCC-CCA or ICC patients, while the AFP >20 ng/ml was more often in the HCC and cHCC-CCA than ICC (all P<0.001).

**Table 1:** Clinical and pathological characteristics of study patients.

Variable	HCC (n = 100)	CHCC-CCA (n = 100)	ICC (n = 100)	P-value
**Mean age (y)** *	57.33 ± 10.85	54.8 ± 11.02	60.06 ± 10.76	0.001
**All patients**				0.24
**Men**	81	74	71	
**Women**	19	26	29	
**Mean nodule size (mm)** *	4.33 ± 3.21	3.82 ± 2.82	4.83 ± 2.81	0.22
**Cause of cirrhosis**				0.608
**Hepatitis B**	94	90	88	
**Hepatitis C**	4	6	6	
**Alcoholism**	2	4	6	
**Tumor maker value**				
**AFP (≥20 ng/ml)**	56	53	14	0.00
**CEA (≥5 ng/ml)**	7	13	14	0.24
**CA 19-9 (≥37 U/ml)**	11	24	42	0.00

Except where indicated, data are numbers of patients.

AFP, a-fetoprotein; CEA, carcinoembryonic antigen; CA 19-9, carbohydrate antigen 19-9; CHCC-CC, combined hepatocellular-cholangiocarcinoma; HCC, hepatocellular carcinoma; ICC, intrahepatic cholangiocarcinoma.

* Data are means ± standard deviations.

### MR Imaging Features

**Table 2** summarizes the MRI features of HCCs, cHCC-CCAs, and ICCs. The three major features—nonrim APHE, non-peripheral washout, and enhancing capsule—were most frequently noted in HCCs (94, 86, and 82%, respectively), followed by cHCC-CCAs and ICCs (all P <0.001). The targetoid mass features—rim APHE, peripheral washout, delayed central enhancement, and targetoid restriction—were most common in ICC (78, 26, 74, and 27%, respectively), followed by cHCC-CCA and HCC (all P <0.001).

**Table 2:** Imaging characteristics of hepatic tumors in cirrhotic liver at gadopentetate acid-enhanced MRI.

Variable	HCC (n = 100)	CHCC-CC (n = 100)	ICC (n = 100)	P-value	Kappa value
**Nodule size**				0.036	
<10 mm	1	5	0		
10-19mm	15	18	10		
≥20 mm	84	77	90		
**Major imaging features**					
Nonrim arterial phase hyperenhancement	95	59	23	0.00	0.87
Nonperipheral washout	88	56	13	0.00	0.88
Enhancing capsule	83	53	24	0.00	0.94
**Targetoid mass imaging features**					
Rim arterial phase hyperenhancement	6	49	77	0.00	0.99
Peripheral washout	1	4	26	0.00	0.98
Delayed central enhancement	7	29	74	0.00	0.83
Targetoid restriction	6	24	27	0.00	0.72
Tumor in the vein	5	11	13	0.14	0.75
**Ancillary imaging features**					
Nodule-in-nodule	3	8	2	0.08	0.85
Mosaic	16	25	6	0.001	0.76
Fat in mass	21	7	1	0.00	0.58
Blood products in mass	17	15	7	0.08	0.89
Peritumoral biliary dilatation	7	30	29	0.00	0.90
Liver surface retraction	13	17	40	0.00	0.92
Corona enhancement	17	23	45	0.00	0.69
Restricted diffusion	99	94	98	0.09	1.0
Mild-moderate T2 hyperintensity	97	94	93	0.42	0.88

Data are numbers of lesions. CHCC-CC, combined hepatocellular-cholangiocarcinoma; HCC, hepatocellular carcinoma; ICC, intrahepatic cholangiocarcinoma.

Fat in mass was more frequently noted in HCC (21% [21 of 100]) than in the other two cohorts (P <0.001), whereas liver surface retraction and corona enhancement were more common in ICC (40% [40 of 100], 43% [43 of 100]) than in the other two cohorts (P <0.001). Compared with HCC, peritumoral biliary dilatation was more common in cHCC-CCA and ICC (30 and 29%, respectively), and the differences were statistically significant (P <.001). Additionally, mosaic architecture-ancillary imaging features favoring HCC were more common in HCC and cHCC-CCA than in ICC (P <0.001). However, the nodule-in-nodule, tumor in vein, and blood products in mass had no significant differences among HCCs, cHCC-CCAs, and ICCs (all P >0.05). Inter-observer agreements for each feature of the LI-RADS categorization are summarized in **Table 2**.

### Diagnostic Performance of LR-5 and LR-M for HCC Versus Non-HCC Malignancies

**Figure 2:**
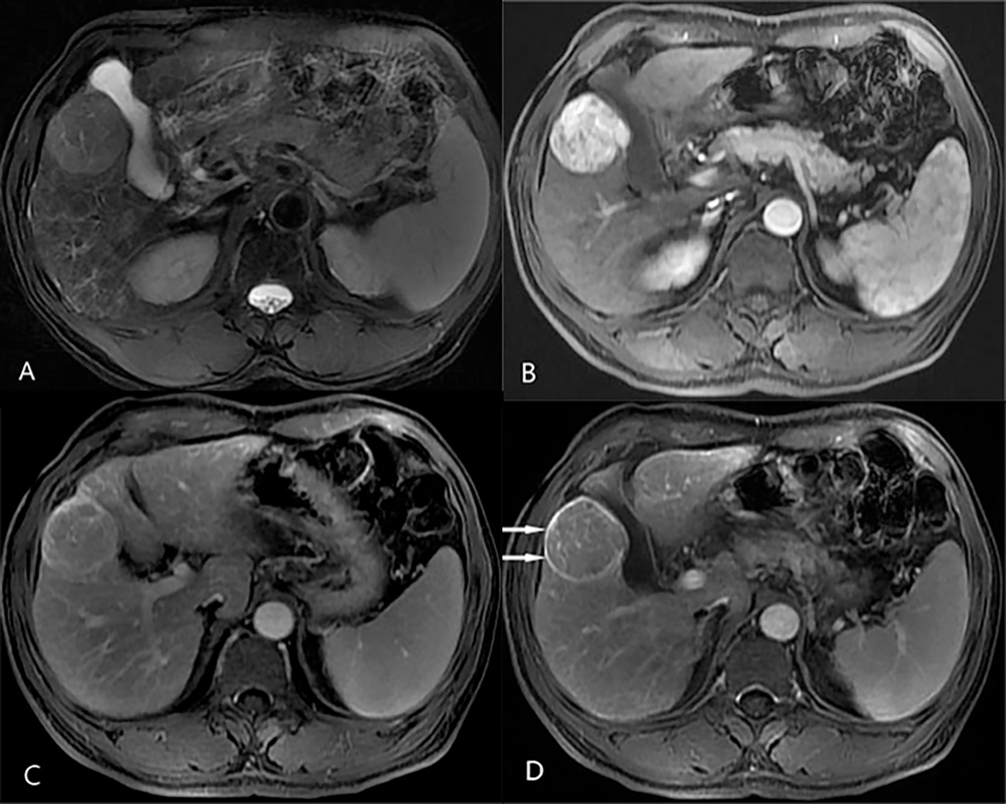
Axial MR images in a 63-year-old man with hepatitis B-related liver cirrhosis and hepatocellular carcinoma that satisfies the LR-5 criteria. **(A)** T2-weighted image shows a 49-mm nodule with mildly homogeneous hyperintensity in hepatic segment V. The nodule shows non-rim hyperenhancement in **(B)** the arterial phase and nonperipheral washout in **(C)** the portal venous phase. An enhancing capsule is also observed in **(D)** the delay phase.

**Figure 3:**
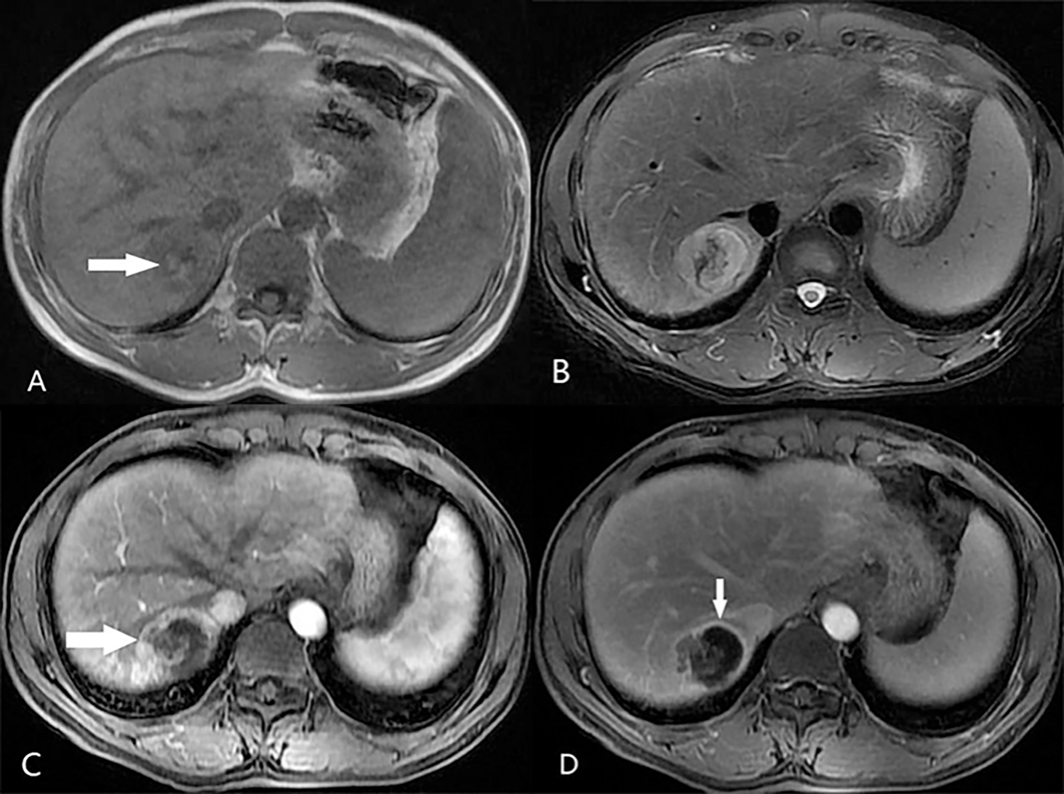
A 48-year-old male patient with hepatitis B-related liver cirrhosis and HCC is categorized as LR-M category. **(A)** T1-weighted image shows a 36-mm nodule with blood product in mass (arrow) in hepatic segment VIII. The nodule shows heterogeneous hyperintensity on **(B)** T2-weighted image. **(C)** The arterial phase shows peripheral enhancement (arrow) on contrast-enhanced T1-weighted imaging with the contrast agent Gd-DTPA, and **(D)** the delay phase shows incompletely enhancing capsule (arrows).

**Figure 4:**
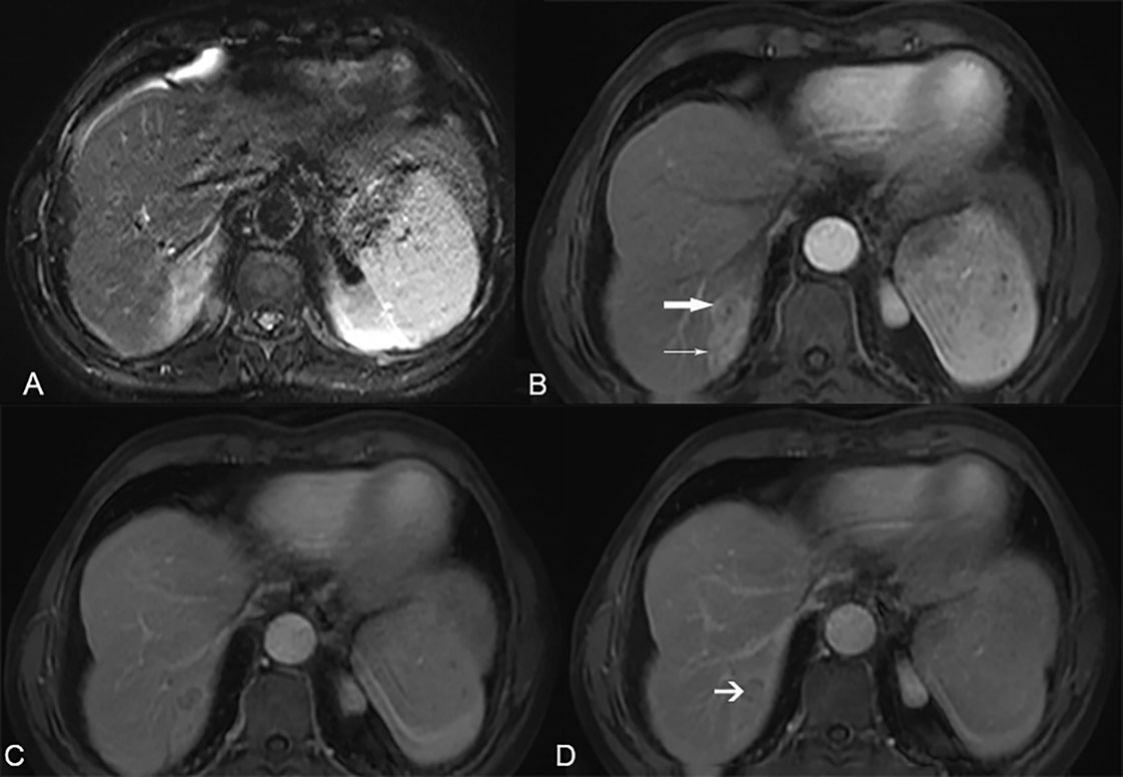
A 61-year-old male patient with hepatitis B-related liver cirrhosis and cHCC-CCA is categorized as LR-M category. **(A)** T2-weighted image shows a 36-mm nodule with heterogeneous hyperintensity in hepatic segment VIII. **(B)** The arterial phase shows peripheral enhancement (arrow) and tumor thrombus in the bile duct (thin arrow) on contrast-enhanced T1-weighted imaging with the contrast agent Gd-DTPA. **(C)** The portal venous phase and **(D)** delay phase show targetoid appearance and the arrow shows progressively delayed central enhancement.

The final LI-RADS categories of the 300 nodules included LR-3 in 8 nodules, LR-4 in 10 nodules, LR-5 in 117 nodules, LR-M in 136 nodules, and LR-TIV in 29 nodules (**Table 3**). In terms of the pathological diagnoses, the LI-RADS categories were inconsistent (P <0.001, **Table 3**): 81% of HCCs, 7% of ICCs, and 29% of cHCC-CCAs were classified as LR-5 (**Figure 2**), and 10% of HCCs, 73% of ICCs, and 53% of cHCC-CCAs were classified as LR-M (**Table 3**) (**Figures 3**, **4**). The sensitivity and specificity of the LR-5 category for differentiating HCCs from other primary liver cancers were 81% (81 of 100) and 82% (164 of 200), respectively. Furthermore, the specificity of the LR-5 category for differentiating between HCCs and ICCs was 93% (93 of 100) and 71% (71 of 100) between HCCs and cHCC-CCAs. Most false-positive diagnoses of HCC were due to 29 of 34 (85%) cHCC-CCAs with LR-5. The sensitivity and specificity of the LR-M category for differentiating non-HCCs (ICCs and cHCC-CCAs) from HCCs were 63% (126 of 200) and 90% (90 of 100), respectively. The sensitivity and specificity of the LR-5 category for diagnosing HCCs in small observations (<20 mm) were 82.5% (33 of 40) and 76.6% (59 of 77), respectively. The LR-5 category had a sensitivity of 82.1% (69 of 84) for diagnosing HCCs, with a specificity of 82.6% (138 of 167). The LR-M category had a sensitivity of 78.3% (18 of 23) for diagnosing non-HCCs (ICCs and cHCC-CCAs), with a specificity of 93.8% (15 of 16). Generally, LR-5 had lower sensitivity and specificity in small observations (<20 mm) than in overall observations. On the contrary, LR-M demonstrated slightly higher specificity and sensitivity for small observations (<20 mm) (**Table 4**).

**Table 3:** Results of LI-RADS categorization of 300 hepatic tumors.

LR-category	All Tumors (n = 300)			Tumor size <20 mm (n = 300)			Tumor size ≥20 mm (n = 300)			P-value
	HCC (n = 100)	CHCC-CCA (n = 100)	ICC (n = 100)	HCC (n = 16)	CHCC-CCA (n = 23)	ICC (n = 10)	HCC (n = 84)	CHCC-CCA (n = 77)	ICC (n = 90)	0
**LR-3**	3	4	1	3 (18.8)	4 (17.4)	1 (10)	0	0	0	
**LR-4**	1	3	6	0	3 (13)	0	1 (1.2)	0	6 (6.7)	
**LR-5**	81	29	7	12 (75)	5 (21.7)	2 (20)	69 (82.1)	24 (31.2)	5 (5.6)	
**LR-M**	10	53	73	1 (6.3)	11 (47.8)	7 (70)	9 (10.7)	42 (54.5)	66 (73.3)	
**LR-TIV**	5	11	13	0	1 (2)	0	5 (6)	11 (14.3)	13 (14.4)	

Liver Imaging Reporting and Data System (LI-RADS) categories are defined as LR-3 (intermediate probability of HCC), LR-4 (probably hepatocellular carcinoma [HCC]), LR-5 (definitely HCC), LR-M (probably malignant, not specific for HCC), and LR-TIV (nodule with definite tumor in the vein). HCC, hepatocellular carcinoma; CHCC-CC, combined hepatocellular-cholangiocarcinoma; ICC, intrahepatic cholangiocarcinoma.

**Table 4:** Diagnostic Performance of LR-5 and LR-M in primary liver cancer according to the size of the observation.

	LR-5		LR-M	
	sensitivity	specificity	sensitivity	specificity
**overall**	81 (81/100)	82 (164/200)	63 (126/200)	90 (90/100)
**The size <20 mm**	75 (12/16)	78.8 (26/33)	78.3 (18/23)	93.8 (15/16)
**The size ≥20 mm**	82.1 (69/84)	82.6 (138/167)	64.7 (108/167)	89.3 (75/84)

Data in parentheses were used to calculate percentages. LI-RADS, Liver Imaging Reporting and Data System.

### Multivariate Analyses of Risk Factors for the MVI of the Primary Liver Cancer

According to the results of MVI-positive, 34 in HCC, 34 in cHCC-CCA, and 30 in ICC (P = 0.006, **Table 5**). In terms of MVI, the LI-RADS categories were inconsistent (P <0.001, **Table 5**): MVI positivity in HCC was mostly classified as LR-5 (61.8%, 21/34); MVI positivity was mostly classified as LR-M in cHCC-CCA (41.2%, 14/34) and ICC (67%, 20/30). Multivariate logistic regression analysis showed that tumor size (odds ratio [OR], 1.255, 95% confidence interval [CI], 1.091–1.444; p = 0.002), peripheral washout (OR, 4.891; 95% CI, 1.437–16.643; p = 0.011), and AFP ≥20 ng/ml (OR, 0.514; 95% CI, 0.291–0.910; p = 0.022) were independent variables associated with the MVI of PLC (**Table 5**).

**Table 5:** Clinical characteristics and multivariate analyses of risk factors for the MVI of primary liver cancer.

Variable	MVI-positive	MVI-negative	P-value	Multivariable Analysis	
	(n = 98)	(n = 202)		OR (95% CI)	P
**Mean age (y)**	57.21 ± 11.52	57.49 ± 10.85	0.279		
**M:F ratio**	73/25	153/49	0.813		
**Mean nodule size (mm)**	5.81 ± 3.63	3.61 ± 2.28	0.000	1.255 (1.091,1.444)	0.002
**Cause of cirrhosis**			0.542		
Hepatitis B	88 (89.8)	184 (91)			
Hepatitis C	7 (7.1)	9 (4.5)			
Alcoholism	3 (3.1)	9 (4.5)			
**Pathology**			0.006		
HCC	34 (34.7)	66 (32.7)			
ICC	30 (30.6)	70 (34.7)			
cHCC-CCA	34 (34.7)	66 (32.6)			
**Tumor maker value**					
AFP≥20 (ng/ml)	50 (51)	73 (36.1)	0.014	0.514 (0.291,0.910)	0.022
CEA≥5 (ng/ml)	14 (14.3)	20 (9.9)	0.261		
CA 19-9≥37(ng/ml)	26 (26.5)	51 (25.4)	0.83		
**Major imaging features**					
Arterial phase hyperenhancement	56 (57.1)	121 (59.9)	0.649		
Non-peripheral washout	53 (54.1)	104 (51.5)	0.673		
Enhancing capsule	51 (52)	109 (54)	0.755		
**Targetoid mass imaging features**					
Rim arterial phase hyperenhancement	47 (48)	85 (42.1)	0.336		
Peripheral washout	4 (4.1)	26 (12.9)	0.017	4.891 (1.437,16.643)	0.011
Delayed central enhancement	33 (33.7)	77 (38.1)	0.454		
Targetoid diffusion restriction	18 (18.4)	39 (19.3)	0.846		
Tumor in vein	20 (20.4)	9 (4.5)	0.000		
**Ancillary imaging features**					
Nodule-in-nodule	5 (5.1)	8 (4)	0.649		
Mosaic	24 (24.5)	23 (11.4)	0.003		
Fat in mass	10 (10.2)	19 (9.4)	0.826		
Blood products in mass	21 (21.4)	18 (8.9)	0.002		
Peritumoral biliary dilatation	31 (31.6)	35 (17.3)	0.005		
Liver surface retraction	34 (34.7)	36 (17.8)	0.001		
Corona enhancement	37 (37.8)	48 (23.8)	0.012		
Diffusion restriction	97 (99)	194 (96)	0.162		
Mild-hyper T2WI	97 (99)	187 (92.6)	0.021		
**LR-category**			0.000		
LR-3	0	8 (4)			
LR-4	1 (1.1)	7 (3.5)			
LR-5	36 (36.7)	81 (40.1)			
LR-M	41 (41.8)	97 (48)			
LR-TIV	20 (20.4)	9 (4.5)			

Data are the number of patients or lesions. Data are presented as count (percentage) and mean ± standard deviation. OR, odds ratio; CI, confidence interval.

Liver Imaging Reporting and Data System (LI-RADS) categories are defined as LR-3 (intermediate probability of HCC), LR-4 (probably hepatocellular carcinoma [HCC]), LR-5 (definitely HCC), LR-M (probably malignant, not specific for HCC), and LR-TIV (nodule with definite tumor in the vein).

## Discussion

Our study evaluated the performance of LI-RADS 2018 for primary liver cancer in the background of liver cirrhosis on MRI combined with extracellular contrast agents based on the updated 2019 WHO classification. The results demonstrated that the sensitivity of the LR-5 category for differentiating HCCs from non-HCCs was consistent with that of the Lee et al. (6) study using LI-RADS 2018 and gadoxetate disodium contrast agent in all observations (81% vs 81%) and small observations (76% vs 75%). Our results had better sensitivity in the LR-5 category at the cost of reduced specificity than that of the previous studies (6, 12, 18, 19), which showed a range of sensitivity between 55.2 and 74%.

It should also be noted that the specificity of the LR-5 category was shown to be lower than that of the recent studies (4, 6, 12, 18, 19) by using LI-RADS v2017 or v2018 with gadoxetate disodium contrast agent. Intriguingly, in small observations (<20 mm), specificity was even lower than that in all observations (78.8% vs 82%). The reason, on one hand, may be the use of hepatobiliary phase imaging and, as shown in the study by Chen et al. (20), significantly higher specificity was observed than in studies of LI-RADS using extracellular contrast agents when the hepatobiliary phase images were added (21).

However, the most common cause of false-positive results was combined HCC-cholangiocarcinoma (cHCC-CCA). In other words, as demonstrated in a recent study (12, 22), cHCC-CCA, which mimics HCC, lowered the specificity of the LR-5 criteria. According to the previous study by Wang et al. (23) with the 2010 WHO classification, more cHCC-CCAs were misclassified as LR-5 with the updated 2019 WHO classification (29% vs 19.5%) in our study. Therefore, we speculated that the updated pathologic diagnosis of biphenotypic PLC in the 2019 WHO classification may impact the LI-RADS categorization of biphenotypic PLCs compared with previous classification systems. Although the result was inconsistent with that of Choi et al. (14), using the gadoxetate disodium contrast agent in patients at risk of HCC, they reported a much higher false-positive rate of LR-5 for cHCC-CCA.

Moreover, compared with the recent study by Yoon et al. (19) with cHCC-CCA in patients with cirrhosis, the rate classified as LR-M in cHCC-CCAs was decreased (58.8% vs 53%). The rate classified as LR-M in cHCC-CCAs was also even lower than that of recent studies (14, 19, 23) in patients at risk. That could explain why LI-RADS v2018 showed decreased specificity in the diagnosis of HCCs than recent reports with LI-RADS v2014 (21) and v2017 (4).

According to our results, LR-M has better specificity for differentiating non-HCCs (ICCs and cHCC-CCAs) from HCCs, which was similar to Lee et al. (6) using gadoxetate disodium contrast agents and LR2018 criteria, than that of the recent studies (10, 12, 18, 24). Particularly, in small observations (<20 mm), LR-M category had better sensitivity and specificity than that in all observations (78.3% vs 63%, 93.8% vs 90%, respectively). We speculate that there are two types of tumors that meet the LR-M criteria: “target sign” and “no target sign.” For tumors<20 mm, the classification of LR-M mainly depends on the target sign. Therefore, the appearance of the target sign necessarily increases the sensitivity and specificity of tumors <20 mm. These results may contribute to risk-based personalized management. Generally, the LR-5 and LR-M category had maintained stable and good specificity and sensitivity in tumor with diameter of ≥20 mm, but the LR-5 category had relatively lower specificity and sensitivity for diagnosing HCC in small observations (<20 mm). Fortunately, the prognosis of most isolated small liver tumors is relatively good.

A previous study by Centonze et al. (25) with a retrospective cohort of 186 patients with HCC undergoing surgery resection reported that LR-5 patients presented with a higher prevalence of MVI, satellitosis, and capsule infiltration, whereas LI-RADS classification did not exert any statistically significant effect on overall survival and relapse-free survival. In our study, although MVI positivity was statistically significantly different in LI-RADS classification, LR classification was not an independent risk factor for predicting MVI in patients with the PLC. We speculated that the possible reason was the heterogeneity of the primary liver tumors. Our results also illustrated that tumor size, AFP ≥20 ng/ml and peripheral washout may indicate a higher risk of the MVI of the PLC. The results were consistent with the previous study by Zhou et al. (26), which showed that larger tumor size, higher AFP level, and CEUS LR-M were significantly correlated with the presence of MVI in HCC (all p <0.05). According to the previous study by Wang et al. (23), the LR-category (P = 0.857) was not effective in predicting MVI of CHCC-CCA preoperatively, which was consistent with our study. The peripheral washout sign was one of the targetoid mass features in category LR-M. The results of MVI in our study may require further prospective studies to verify.

Our study also has several limitations. First, coincidentally, since the ICC cohort had the same number of cases as the cHCC-CCA cohort, we conducted our study retrospectively at random in the same number. It can lead to selection bias, where the proportion of ICC and cHCC-CCA is overestimated compared to the actual incidence of cirrhosis. Second, there were only 49 small observations (<20 mm), as many small HCCs were treated through local–regional treatment after having noninvasive diagnosis based on imaging features in our institution. Finally, we did not evaluate visibility at screening US and threshold growth, which might have caused an alteration of the sensitivity of LR-5 in the diagnosis of HCC.

In conclusion, our study demonstrates that the LR-5 category of Liver Imaging Reporting and Data System (LI-RADS) version 2018 based on the updated 2019 WHO classification with enhanced MRI with extracellular contrast agent had low specificity, particularly in small observations (<20 mm). Additionally, the LR-M category can effectively differentiate non-HCC malignancy from hepatocellular carcinoma (HCC) in cirrhosis.

## Data Availability Statement

The original contributions presented in the study are included in the article/supplementary material. Further inquiries can be directed to the corresponding authors.

## Ethics Statement

The studies involving human participants were reviewed and approved by The Institutional Review Board of Zhongshan Hospital, Fudan University. The patients/participants provided their written informed consent to participate in this study. Written informed consent was obtained from the individual(s) for the publication of any potentially identifiable images or data included in this article.

## Author Contributions

XL had full access to all of the data in the study. XL, CM, CZ, and YW took responsibility for the integrity of the data and the accuracy of data analyses. YL and CZ designed the study. Due to computer spelling error, here is changed to YL, CY and YW conducted the analyses and interpreted the data. YL, XNL, and XN drafted the manuscript and have contributed equally to this work and share first authorship. All authors listed have made a substantial, direct, and intellectual contribution to the work and approved it for publication.

## Funding

This study was supported by the Clinical Research Plan of SHDC (grant number SHDC2020CR1029B), the National Natural Science Foundation of China (grant number 82171897), the Shanghai Municipal Key Clinical Specialty (grant number shslczdzk03202), the Clinical Research Project of Zhongshan Hospital, Fudan University (grant number 2020ZSLC61), and the Henan Provincial Universities Key Scientific Research Projects (grant number 21B320002).

## Conflict of Interest

The authors declare that the research was conducted in the absence of any commercial or financial relationships that could be construed as a potential conflict of interest.

## Publisher’s Note

All claims expressed in this article are solely those of the authors and do not necessarily represent those of their affiliated organizations, or those of the publisher, the editors and the reviewers. Any product that may be evaluated in this article, or claim that may be made by its manufacturer, is not guaranteed or endorsed by the publisher.
